# Methods to evaluate driving competence for people with acquired brain injury (ABI): A systematic review

**DOI:** 10.3389/fresc.2022.1020420

**Published:** 2023-01-04

**Authors:** Doha Alhashmi, Aislinn Lalor, Ellie Fossey

**Affiliations:** ^1^ Department of Rehabilitation, College of Health and Rehabilitation Sciences, Princess Nourah Bint Abdulrahman University, Riyadh, Saudi Arabia; ^2^ Department of Occupational Therapy, School of Primary and Allied Health Care, Faculty of Medicine, Nursing and Health Sciences, Monash University, Frankston, Victoria, Australia; ^3^ Rehabilitation, Ageing and Independent Living Research Centre, School of Primary and Allied Health Care, Monash University, Frankston, Victoria, Australia; ^4^ Living with Disability Research Centre, School of Allied Health, La Trobe University, Melbourne, Victoria, Australia

**Keywords:** driving assessment, driving fitness, readiness to drive, brain injury, driving screening, occupational therapy

## Abstract

Driving is essential for independence, community involvement and quality of life. Driving is the primary transportation method in Saudi Arabia. Despite the high rates of brain injuries and disability in Saudi Arabia, currently there are no guidelines regarding driver assessment and rehabilitation to facilitate people with brain injuries to resume driving. Therefore, this systematic review aimed to understand the assessment methods used internationally to evaluate driving competence for people with acquired brain injuries (ABI). A systematic search of six electronic databases was conducted by two authors and twenty-six studies were identified for review. Four main approaches to driver assessment: clinical assessments such as neuropsychological tests, off-road screening tools, simulator testing, and comprehensive driving assessment were identified. However, our findings revealed a lack of consistency in their use to assess driving competence after ABI. On-road driving performance tests were predominantly used to determine driving competence either independently or in combination with another method in over two-thirds of the reviewed studies. While clinical assessments of cognitive impairments showed some capacity to predict driving performance of people with ABI, they should be used with caution since they cannot replace on-road driving performance tests. Driver assessment should be part of rehabilitation following high prevalence conditions such as ABI. This systematic review offers guidance for Saudi clinicians, as well as policymakers, about providing rehabilitation services for people with ABI, and recommendations for further research and collaborations to improve this much-needed area of practice.

## Introduction

Driving is an activity that supports people's independence and community involvement (1). Having a driver's license is important as it enables people to participate in valued daily activities (2, 3). Driving status and mobility affects physical and psychological health, as well as social participation and life satisfaction. Driving cessation conversely is related to decrease social participation, depression and isolation (4, 5).

In the Kingdom of Saudi Arabia (KSA), motor vehicles are considered the primary means of transportation within and between cities (6). Given driving is essential to many aspects of daily life for most people in Saudi Arabia, resuming driving is a priority for many patients following major injury for occupational, socio-economic and socio-cultural reasons (17). Traumatic brain injury (TBI) and stroke are leading causes of disabilities in Saudi Arabia (7). Driving is a complex activity that requires a variety of critical abilities such as cognitive, motor, perceptual, and executive functions, many of which could be impaired following acquired brain injuries (ABI) (10). Therefore, the ability to return to driving safely is of particular concern in this population.

People with brain injuries are reported to have a higher crash rate in comparison with the base rate of crashes in the United States general population (11), and between 58%–80% of stroke patients are not able to return to drive (12, 13). Similarly, a multicentre study in the United Kingdom reported only 36.5% of people resume driving following head injury (14). Moreover, multiple research studies report clients returning to driving without medical clearance (3, 15, 16). The situation is less well understood in Saudi Arabia, but a recent study reported only 7 out of 94 patients resumed driving post stroke and none had undergone any form of driving assessment or received driving-related information (17).

There is international agreement on the importance of assessing the driving competence of people considered medically at-risk drivers. Driving competency as defined by the Transportation Research Board (2016) is “the demonstration of fitness to drive that meets criteria recognized by a body responsible for driver licensing” (18, *p*.6). In Australia, the United Kingdom, Canada and the United States, published medical guidelines regarding driving assessments are available to regulate the medical practice and inform health professionals including physicians, occupational therapists, and optometrists (19–21). In comparison, there is no structured guideline to facilitate the process of returning to driving safely for people with disability in Saudi Arabia (17). Yet, the high rates of stroke and traumatic injuries in Saudi Arabia mean that a growing number of potentially medically at-risk drivers need assessment to enable safe driving.

Several systematic reviews and meta-analyses have been completed to facilitate evidence-based decision-making regarding driving assessment methods following stroke or traumatic brain injuries (TBI) (2, 22-25). In terms of assessing driving competence, Classen et al. (2009) (2) reviewed assessment methods used following severe, moderate and mild TBI. This review extracted data from 13 studies and classified the assessment methods used to assess driving competence following TBI. The review authors classified them as neuropsychological, simulator, off-road, self-report, and comprehensive driving evaluation (2). However, regardless of the assessment methods used, the authors concluded that there was no consistency on reporting the level of TBI severity within the included studies. Thus, recommendations on what methods to use in assessing driving competence were not generalizable to clients with different TBI severity levels (2). Systematic reviews by Baker, Unsworth, and Lannin (2015) (22) and Egeto, Badovinac, Hutchison, Ornstein, and Schweizer (2019) (23) attempted to address the gap presented by Classen et al. (2009) (2) *via* investigating driving competence with mild TBI separately from moderate and severe TBI. Both of these reviews identified a lack of consistent and standardised guidelines on competence to drive following TBI (22, 23). Other reviews by Devos et al. (2011) (24) and Hird, Vetivelu, Saposnik, and Schweizer (2014) (25) focused on driving assessment methods used following stroke. Devos et al. (2011) (24) reviewed clinical assessments, while Hird et al. (2014) (25) looked broadly into clinical, simulators and on road assessment methods. These two reviews reached similar conclusions about the heterogeneity of methods used to assess fitness to drive after stroke.

Given the lack of available guidelines in Saudi Arabia, the present systematic review focuses on identifying the available international evidence about methods of assessing driving competence following ABI, including both stroke and TBI. This evidence may then be used to inform decision-making about driving competence in rehabilitation policy and practice within Saudi Arabia.

## Methods

This systematic review is reported according to the Preferred Reporting Items for Systematic Reviews and Meta-Analyses (PRISMA) checklist (26), however, the protocol was not registered for this review. The research question is: What assessment methods inform the evaluation of driving competence following ABI, including TBI and stroke?

### Information resources

Six electronic databases including: CINAHL (Cumulative Index to Nursing and Allied Health Literature) Plus, Transportation Research Information Database (TRID), Ovid Emcare, Ovid MEDLINE, Ovid Embase, and PsycINFO, were searched on 20th March 2019. A follow up search was conducted on 2nd August 2021 to identify any additional eligible publications.

### Eligibility criteria

Articles were eligible for inclusion in this systematic review if each of the following criteria were satisfied: i) the population included in the study had sustained any type of ABI, including participants with other conditions as long as participants with ABI were included; and ii) the assessment methods typically used in driving assessment practice had been included. For the purpose of this review, these assessment methods are categorized as: a) Clinical assessments including neuropsychological tests, and off-road screening tools, b) Simulator testing, and c) Comprehensive driving assessment. Also, iii) articles were included if they reported peer-reviewed original research studies. Articles were excluded if one or more of these criteria were identified: i) data for the ABI group were not reported separately or not able to be separated from other participant groups; ii) study designs were either opinion-based articles, narrative case studies, or assessments based on self-report or feedback of significant others; or, iii) they were not available in English. Additionally, only studies published after 1994 were included since research in the driving field has mainly been shaped after the mid-1990s (2).

### Search strategy and study selection

The search strategy comprised combinations of free-text terms and thesaurus (e.g., MeSH) terms relating to assessments of fitness to drive for people with ABI and was developed by the primary author (DA) with the help of a specialist tertiary librarian.

**Figure 1:**
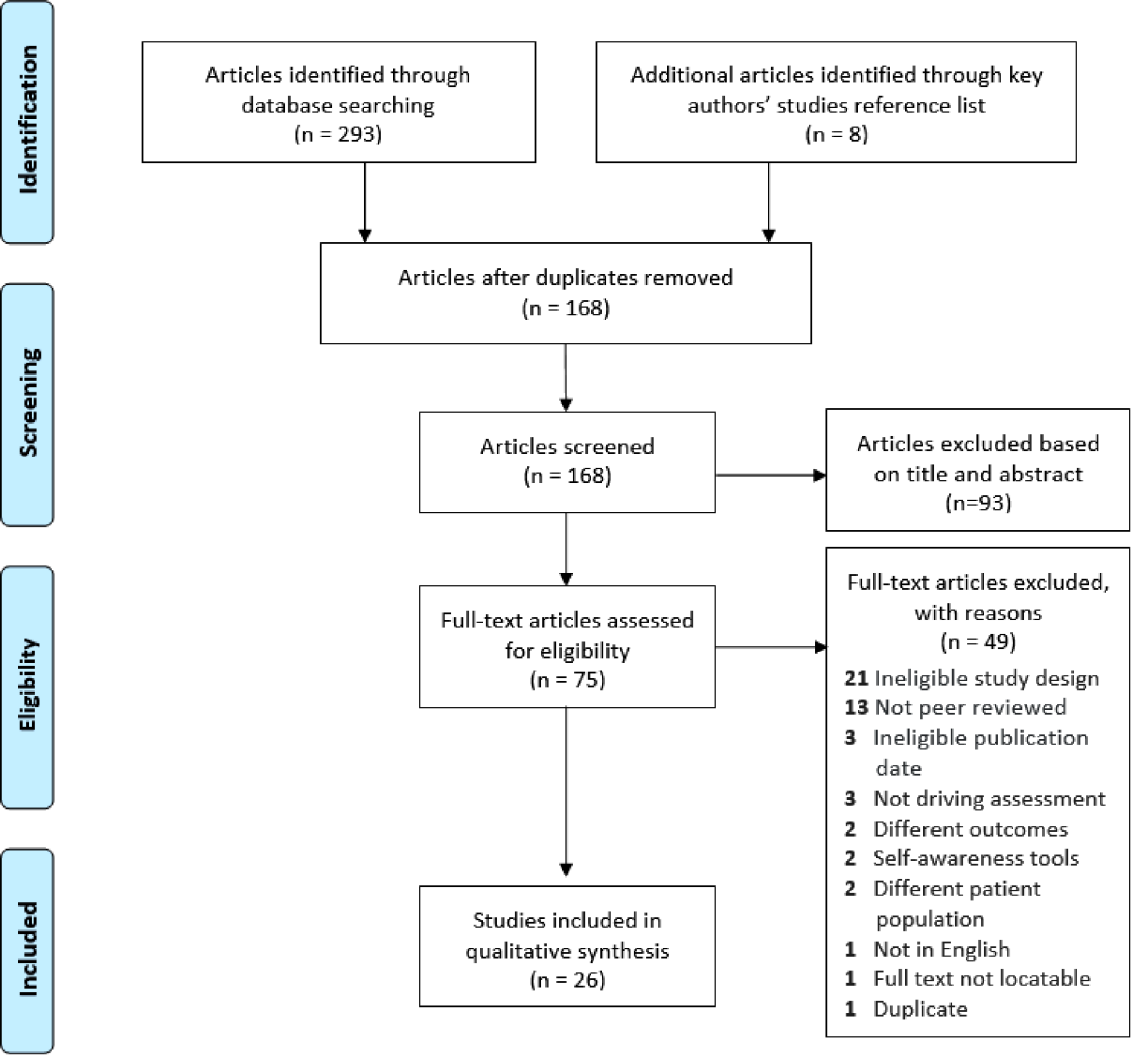
Flowchart of the systematic literature search.

The initial search strategy was developed for and used in Ovid MEDLINE, and was then adapted and replicated for use in the other five databases (see Supplementary file). Keywords included automobile driv*; OR automobile driver examination; OR driv* assess*; OR fit* drive*; OR driv* abilit*; OR read* driv*; OR driv* test; OR driv* rehab*; combined with the keywords brain injur*; OR stroke; OR traumatic brain injur*. (Refer to the Supplementary file for search strategy). The initial search identified 301 citations which were downloaded to a bibliographic management software (EndNote version 8), and 133 duplicate studies were removed as shown in the flow chart (see Figure 1). Two authors (DA and AL) completed title and abstract screening using systematic review software, Covidence to assess eligibility for inclusion in the systematic review. Consensus for inclusion of a study was reached through agreement between the two authors and where agreement could not be reached, a third author (EF) was consulted. On the 7 studies where DA and AL differed in their initial appraisal of eligibility, consensus was reached for 5 studies through discussion and the third author was consulted to reach consensus on 2 studies. This resulted in 75 articles for full text screening. Two authors (DA and AL) completed full text screening and reviewed the reference list of articles from key authors in the field resulting in 26 studies that met the inclusion criteria for the review. Articles were excluded predominantly due to ineligible study design.

### Quality assessment

Quality assessment of eligible studies was independently completed by two authors (DA and AL) using a modified version of the Downs and Black Instrument (27).

The Downs and Black Instrument (1998) is a checklist for the assessment of the methodological quality of randomised and non-randomised studies comprising 27 items with Yes, No, or Not Applicable as response options. A score of 1 is granted for each Yes. In the unmodified Downs and Black Instrument, scores range from 0 to 34, where 34 indicates a study is high quality and 0 indicates low quality. Items are grouped to measure: i) reporting, ii) internal validity, iii) external validity, and iv) power. The checklist has acceptable face, content validity, and internal consistency (KR-20: 0.89), test–retest reliability (r 0.88), and inter-rater reliability (r 0.75) (27). Moreover, the Downs and Black Instrument can be modified to suit the requirements different research questions (27). Thus, modifications were made in a similar way to that described by Baker et al. (2015) (22), with six items (4, 8, 14, 19, 23, and 24) eliminated because they relate to intervention studies which were not applicable to the present systematic review as this review includes a population (people with ABI including TBI and stroke) and outcome (driving competence). Item 27 was also modified to Yes or No to assess the study power, as has been done previously in other studies (28). For the purpose of this study, the maximum score was 22 instead of 34.

Two authors (DA and AL) also reviewed all included studies for their level of evidence to ensure trustworthiness and creditability. The Oxford Centre for Evidence Based Medicine-Level of Evidence, in which the highest level of evidence is 1, and the lowest level of evidence is 5, was used for this purpose in this review (29).

### Data extraction

Two authors (DA and AL) independently completed data extraction for all 26 studies meeting eligibility for inclusion. Data extracted for each study included: i) the citation (authors, publication year, title, journal, article type); ii) demographics (sample size, age of participants, gender ratio, study location, participant condition, time after injury, driving experience); iii) study methods (aim(s), design, assessment measure used to assess or screen fitness to drive, components measured by the assessment, simulator used or not, who conducted the assessment, clinical/on-road assessment conducted); and iv) results (ability to determine or inform competence to drive decision, overall results, potential sources of bias).

### Data analysis

When planning this systematic review, it was anticipated that this systematic review could include a wide variety of study designs and competence to drive assessment methods and outcome measures that might not be sufficiently homogenous for quantitative synthesis. Thus, the data were descriptively categorised by assessment type: a) Clinical assessments including neuropsychological tests, and off-road screening tools, b) Simulator testing, and c) Comprehensive driving evaluation (CDE) which is defined as “clinical assessment using tools that correlate with driving performance or crash, followed by on-the-road assessment” (2) *p*.582.

## Results

### Descriptive profile of the primary studies

In total, 26 studies were identified for review, with publication date ranging between 1997 and 2019. Included studies were conducted in eight different countries, however over 70% of the included studies were conducted in Unite States, Australia and Sweden. Sample sizes varied from 26 to 269 participants with predominantly male participants (58%) and a mean age of 54.5 years where the youngest participant's age was 16 years old and the oldest was 93 years old (see Table 1). As Table 1 shows, the most studied population (*n* = 9) was the stroke population, while five studies investigated only people with TBI. In five other studies, both stroke and TBI were investigated under the umbrella of ABI; while the remaining seven studies comprised mixed population with various neurological conditions. The time since injury onset was reported in 20 studies and varied between 24 h to 7 years.

**Table 1:** Participant’s characteristics.

Reference	Etiology (*n*.)	N (% Male)	Age *mean (SD), range in* years	Driving experience *mean (SD)* in years	Time after Injury mean (SD) or range in months
Akinwuntan et al. (2002) (31)	Stroke	104 (78.8)	56.8 (11.9), 30–79	34.9 (12.4)	18.5 (20.0)
Akinwuntan et al. (2006) (32)	Stroke	68 (83.8)	53.0 (13.0)	33.0 (13.0)	15.0 (18.0)
Akinwuntan et al. (2013) (33)	Stroke (15)	31 (61.3)	52.0 (12.0)	34.0 (9.0)	5.0 (2.0)
	Control (16)		40.0 (16.0)	23.0 (16.0)	
Baker, Unsworth, & Lannin (2015) (34)	TBI (60)	120 (75.0)	39.5 (15.4), 18–64	20.3 (15.3)	24 hrs. and 2 weeks
	Ortho (60)		38.2 (13.3), 19–65	9.3 (13.4)	
Bliokas, Taylor, Leung, & Deane (2011) (35)	Stroke (61)	104 (76.9)	61.4 (16.7), 17–93	39.7 (16.4)	3.0–4.0
	TBI (14)				
	Dementia (8)				
	Parkinson’s disease (6)				
	Other neurological conditions (15)				
Caneman & Panzitta (1997) (30)	Stroke (15)	45 (not stated)	(not stated)	(not stated)	3.0–9.0
	Control (30)				
Cullen, Krakowski, & Taggart (2014) (36)	TBI	38 (78.9)	(not stated)	(not stated)	(not stated)
		Drivers: 19	48.5 (14.3)		
		Non-drivers: 19	49.0 (14.9)		
Duquette et al. (2010) (37)	Stroke (95), TBI (92)	187 (76)	(not stated)	(not stated)	(not stated)
		Centre 1: 111	55.4 (18.4)		
		Centre 2: 76	48.1 (14.3)		
Elgin et al. (2010) (38)	Normal vision (30)	60 (48.3)	52.0 (19.0)	(not stated)	Less than 6.0
	BI/Hemianopia (22)		52.0 (20.0)		
	BI/Quadrantanopia (8)		55.0 (22.0)		
Esser et al. (2016) (39)	BI (17)	135 (not stated)	45.4 (18.0)	(not stated)	(not stated)
	Dementia (50)		74.9 (12.7)		
	Stroke (39)		67.7 (13.4)		
	Parkinson’s disease (13)		66.7 (10.0)		
	MS (16)		60.1 (19.3)		
George & Crotty (2010) (40)	Stroke	66 (78.8)	65.9 (8.4)	47.3 (not stated)	10–2,190 days
George, Clark, & Crotty (2008) (41)	Stroke	26 (92.3)	65.6 (13.2)	46.3 (13.4)	21.0–816.0 days
Hargrave, Nupp,& Erickson (2012) (42)	Stroke (48), TBI(28)	76 (76.0)	57.3 (17.0), 18.0–87.0	(not stated)	(not stated)
Hartman-Maeir, Erez, Ratzon, Mattatia, & Weiss (2008) (43)	Stroke (17)	30 (80.0)	57.9 (18.0), 20–80	33.1 (15.5)	22.27 (21.99)
	TBI (9)				
	Anoxic brain injury (4)				
Korner-Bitensky et al. (2000) (44)	Stroke	269 (80.0)	63.6 (12.5)	(not stated)	6.9 (11.0)
McKay, Liew, Schonberger, Ross, & Ponsford (2016) (45)	TBI	99 (86.0)	40.6 (14.8), 18–74	20.5 (15.1)	7.0 (6.0)
Novack et al. (2006) (46)	TBI	60 (36.3)	33.0, 16–68	(not stated)	2 months to 19 years
Patomella, Caneman, Kottorp, & Tham (2004) (47)	Stroke (28), TBI (3)	31 (70.9)	57.0 (12.2), 22–77	(not stated)	7.0
Patomella, Tham, & Kottorp (2006) (48)	Stroke	101 (87.1)	61.9 (10.1)	(not stated)	13.4 (13.9)
Patomella, Tham, Johansson, & Kottorp (2010) (49)	Stroke (128)	205 (84.0)	69.0 (11.0), 33–86	(not stated)	(not stated)
	MCI (43)				
	Dementia (34)				
Samuelsson, Modig-Arding & Wressle (2018) (50)	Stroke (134)	204 (77.0)	60.0 (12,7), 23.0–86.0	(not stated)	(not stated)
	TBI (20)				
	MCI (30)				
	Tumours (8)				
	Cerebral infection (3)				
	Cognitive impairment due to a neurological disease like MS, Parkinson’s Disease (9)				
Schanke & Sundet (2000) (51)	Stroke (43)	55 (76.4)	56.1 (13.3), 20–80	(not stated)	27.6
	TBI (5)				
	MS (4)				
	Other (3)				
Schneider & Gouvie (2005) (52)	TBI (40)	80 (40.0)	21.9 (4.0)	(not stated)	7.13 (5.08) years
	Control (40)		21.9 (3.9)		
Schultheis, Hillary, & Chute (2003) (53)	ABI (15)	30 (63.3)	38.6 (10.8), 21–59	21.0 (9.7)	23.8 (22.8)
	Control (15)		33.2 (6.8), 23–45	16.2 (6.6)	
Selander, Johansson, Lundberg, & Falkmer (2010) (54)	Stroke (76)	195 (86.1)	65.3 (9.8), 43–85	(not stated) (not stated)	6
	Cognitive deficits (119)		72.2 (9.3), 47–88		
Unsworth et al. (2019) (55)	Stroke	148 (67.6)	65.3 (14.7), 20–95	42.7 (16.2)	(not stated)

ABI, acquired brain injury; BI, brain injury; MCI, mild cognitive impairment; MS, multiple sclerosis; Ortho, orthopedical conditions; TBI, traumatic brain injury.

Study designs included 24 cohort studies, one case control and one unspecified quantitative study (see Table 2). Nearly all included studies had level 4 evidence using the Oxford Centre for Evidence Based Medicine- Level of Evidence (29) except for one where sufficient information to determine the level of evidence was unavailable (30). Small sample size was one of the most common methodological limitations amongst the included studies. Use of a retrospective approach contributed to sample heterogeneity around demographic, driving history and injury related variables in some studies. For example, driving experience was not reported in 9 of the included studies, whereas the other 17 studies measured it in different ways. Table 2 provides a summary of characteristics of each study included in this systematic review.

**Table 2:** Summary of studies included in the systematic review.

Author(s) (Year)	Design and Location	Purpose	Assessment tool used	Outcome Variable	Main Findings	Quality Rating
Akinwuntan et al. (2002) (31)	Retrospective cohort Brussels, Belgium	To identify predictors of team’s decision in regards to driving performance in stroke patients.	Mono- and binocular vision, stereoscopy, figure of ray; RCFT; UFOV; Fimm-Zimmermann test battery	Multi-professional team decision and on-road driving test performance	On road testing was the determining factor (R^2^ .42)	16/22
Akinwuntan et al. (2006) (32)	Prospective cohort Brussels, Belgium	To identify a battery assessments that that could predicts driving performance after stroke.	Mono- and binocular visual acuity tests; test of kinetic vision; RCFT; UFOV; 5 tests from the TAP battery: divided attention, visual scanning, incompatibility, visual field, visual neglect; component tests of the SDSA	Multi-professional team decision	The best model to predict the team decision was a combination of visual neglect, RCFT, and on-road test.	14/22
Akinwuntan et al. (2013) (33)	Prospective cohort Augusta, United States	To investigate the ability of the US version of the Stroke Driver Screening Assessment (SDSA) to predict driving performance after stroke.	SDSA	Driving performance on high fidelity driving simulator	Simulated driving performance of participants with stroke was predicted with 87% accuracy. Healthy participants’ simulated driving performance was predicted with 88% accuracy.	16/22
Baker, Unsworth, & Lannin (2015) (34)	Quasi-experimental case-control Melbourne, Australia	To investigate driving competence of patients with mTBI in the acute stages.	OT–DHMT; Road Law and Road Craft Test; University of Queensland-Hazard Perception Test; MMSE	Self-reported driver status after follow up period.	OT–DHMT was the only tool that presented a significant difference (*P *= .01) between mTBI and orthopaedic scores; those who took longer to complete the maze were most likely to fail the on-road assessment (*P *= .06).	20/22
Bliokas, Taylor, Leung, & Deane (2011) (35)	Prospective cohort Wollongong, Australia	To investigate a neuropsychological assessment battery to assess driving competence in individuals with cognitive impairment.	BD; DS; JLO; PA; RAVLT; RCFT; TMT-A; VFD; WCST	On-road driving performance	With 73% sensitivity and 76% specificity The battery was able to classify on road tests outcome (pass/fail). None of the individual tests were solely able to predict the on road driving performance outcome.	17/22
Caneman & Panzitta (1997) (30)	Quantitative unspecified Västerås, Sweden	To investigate the acceptability of using a simulator to determine driving competence after stroke	Driving simulator	The acceptability of stimulator	No statistical analysis. Out of 15 participants, 13 acknowledged that the simulator is useful and that it felt real.	8/22
Cullen, Krakowski, & Taggart (2014) (36)	Retrospective, matched cohort Toronto, Canada	To investigate the predictive ability of neuro-psychometric tests when relating to return to driving after TBI	TMT-A; TMT- B; Digit Span–forward and backward	Return to driving, as defined by reinstatement of driver’s licence, through paper questionnaire	TMT-A and TMT-B scores were significantly better for those who returned to drive. TMT-A (*P *= .01); TMT-B (*P *= .01).	20/22
Duquette et al. (2010) (37)	Retrospective cohort Montreal, Canada	To determine if the partial administration of the CBDI could affect its concurrent validity	CBDI	On-road Driving performance	Partial administration of the CBDI, can affect its concurrent validity. However, even when administered completely did not classify more than %50 of those with CVA or TBI who failed the on-road test.	16/22
Elgin et al. (2010) (38)	Prospective cohort Birmingham, Alabama, United States	To determine whether drivers with quadrantanopia or hemianopia when compared with drivers with normal visual ﬁelds could be safe drivers on the road.	Comprehensive eye examination Automated static perimetry; MMSE; DS of WAIS; TMT-A; TMT-B	On-road driving performance	A good performance in clinical tests including: contrast sensitivity, average visual ﬁeld sensitivity in the intact ﬁeld, and TMT-A was associated with the potential safe driving on road. Contrast sensitivity (*P *= .003); average visual ﬁeld sensitivity in the intact ﬁeld (*P *= .016); TMT-A (*P *= .0036).	14/22
Esser et al. (2016) (39)	Retrospective cohort Birmingham, Hull, Cannock, Oxford, Worcester, Northampton, Leamington, UK	To determine the predictive ability of the MOCA as a screening tool to predict pass/fail of on-road testing for people with cognitive concerns.	MOCA	On-road driving performance	There was a signiﬁcant difference in total MOCA scores (*P* < .001) with cut-offs of >27 indicating that the participant is more likely to pass the on-road test; while scores of MOCA <12 were most likely to fail the on-road test.	14/22
George & Crotty (2010) (40)	Prospective cohort Adelaide Australia	To examine the UFOV and SDSA’s criterion validity through comparison to on-road performance outcome.	UFOV and SDSA	On-road driving performance	There was significant results from 2 UFOV subtests (Divided Attention subtest, *P *< .01; Selective Attention subtest, *P *< .05) as well as results from SDSA (*P *< .05) in relation to on-road test recommendations. The highest sensitivity value goes to the divided attention subtest of the UFOV (88.9%).	17/22
George, Clark, & Crotty (2008) (41)	Prospective cohort Adelaide, Australia	To determine the VRST construct and predictive validity	VRST; VSA; RTM	On-road driving performance	VRST scores were associated with on-road test outcome, predicting those who would pass from those who would be required some more driving lessens to pass .	16/22
Hargrave, Nupp, & Erickson (2012) (42)	Retrospective cohort Colorado, United States	To determine the predictability of FAB and TMT-B of the on-road test outcome for individuals with stroke or traumatic brain injury	FAB and TMT-B	On-road driving performance	Only the scores from TMT-B significantly predicted the on-road driving performance (*P *= .05) with a cut-off score of 90 seconds or more classifying 77% of those failing the on-road test.	20/22
Hartman-Maeir, Erez, Ratzon, Mattatia, & Weiss (2008) (43)	Retrospective cohort Tel Aviv, Israel	To examine the validity of the CTT as a clinical assessment of driving competence of individuals with ABI.	CTT and UFOV	On-road driving performance	CTT performance time cut offs scores below 60 seconds indicated passing the on-road test; scores over 60 seconds indicated failing the on-road test. While UFOV divided and selective attention subtests significantly related to on road pass/fail outcome.	19/22
Korner-Bitensky et al. (2000) (44)	Retrospective cohort New York, Brunswick, Indiana, Wisconsin, United States; Quebec, Canada	To determine the predictive ability the MVPT for on-road test outcome in individuals with stroke.	MVPT	On-road driving performance	MVPT scores of less than or equal 30 indicates poor visual-perception; while >30 indicates good visual perception. The predictive validity of MVPT is not sufficient, as the positive predictive value was 60.9% (*n* = 67/110) and the negative predictive value was 64.2% (*n* = 102/159).	16/22
McKay, Liew, Schonberger, Ross, & Ponsford (2016) (45)	Retrospective cohort Melbourne, Australia	To examine the correlations between clinical cognitive tests and on-road performance outcome in individuals with TBI.	The Wechsler Test of Adult Reading, RAVLT, RCFT, BD, Similarities subtest, Digit Span subtest, DS and SDMT from (WAIS-III), TMT-B	On-road driving performance	Cognitive tests were found to have week correlation and poor prediction to on-road test outcome (r values <0.3).	21/22
Novack et al. (2006) (46)	Prospective cohort Alabama, United States	To examine the relationship between UFOV and on-road driving performance outcome in individuals with TBI	UFOV, TMT-B, braking reflex test	On-road driving test performance	Significant relationship between UFOV divided attention subtest and on-road test outcome (*P *< .05).	11/22
Patomella, Caneman, Kottorp, & Tham (2004) (47)	Prospective cohort Solna, Sweden	To evaluate the validity of P-Drive	P-Drive	P-Drive to evaluate simulated driving performance; Rasch analysis of P-Drive.	Goodness-of-fit was demonstrated by all P-Drive items.	11/22
Patomella, Tham, & Kottorp (2006) (48)	Prospective cohort Solna, Sweden	To investigate some aspects of P-Drive’s validity and stability when used in a simulator.	P-Drive	P-Drive to observe simulated driving performance; Rasch analysis of P-Drive.	P-Drive items demonstrated acceptable goodness-of-fit (95%) and participants demonstrated acceptable goodness-of-fit (97%).	12/22
Patomella, Tham, Johansson, & Kottorp (2010) (49)	Prospective cohort Solna, Sweden	Determine P-Drive’s internal scale validity and reliability when used on-road for individuals with neurological disorders.	P-Drive	P-Drive to observe on-road driving performance; Rasch analysis of P-Drive.	P-Drive person response validity was within acceptable level. P-Drive is able to differentiate between the driving abilities of people with neurological disorders with a person separation reliability of 0.90.	12/22
Samuelsson, Modig-Arding & Wressle (2018) (50)	Retrospective cohort Sweden	To examine the predictive value of some assessment tools used by occupational therapist to inform their decision regarding driving competence following cognitive impairments due to brain disease or trauma.	SDSA; UFOV; Driving simulator; On-road driving performance observation notes.	Multi-professional team decision	The UFOV presented the highest sensitivity of all measures (78%). Moreover the combination of UFOV and the results from the simulator had even a higher sensitivity was 87%. While the specificity of predicting the team decision for both simulator and UFOV was only (55%).	16/22
Schanke & Sundet (2000) (51)	Retrospective cohort Alværn, Norway	To determine the relationship between a verity of neuropsychological tests results and on r-road test outcome.	Friedman Visual Field Analyzer Mk2; Digit Span; Grooved Pegboard; TMT-A; TMT-B; SDMT (Oral); Similarities; Picture Completion; BD Copy-a-cross (3d); SNST; Awareness Index	On-road driving performance	To differentiate between the groups’ driving performance (awareness of cognitive impairments, visuo-constructive, reaction time, and visual attention) were found to be important.	18/22
Schneider & Gouvie (2005) (52)	Prospective cohort Louisiana, United States	To examine the ability of the UFOV to predict accident involvement of those with mTBI	UFOV; TMT-A; TMT- B; WAIS-III; Processing speed index; SDMT	Self-reported survey of the number of accidents and traffic citations received during the past 2 years	T-test of independent sample showed no significant difference in any of the measures (all *P *> .05); and the UFOV was not able to predict driving abilities of individuals with mTBI.	16/22
Schultheis, Hillary, & Chute (2003) (53)	Prospective cohort with matched control New Jersey, United States	To compare NDT with a comprehensive hospital-based driving evaluation in assessing driving competence for individual with ABI. As well as contrast the performance on NDT between ABI individuals who passed the on-road test and those who failed with control participants.	NDT	Comprehensive hospital-based evaluation and on-road driving test performance	NDT was able to classify 80% of all participants with ABI with a statistically significant Spearman correlation (*r* = .743, *P *< .01).	14/22
Selander, Johansson, Lundberg, & Falkmer (2010) (54)	Prospective cohort Stockholm, Sweden	To determine the SDSA predictability of the on-road test outcome for individuals with stroke and cognitive deficits/dementia.	SDSA	On-road driving test performance	SDSA is not predictive of the on-road test outcome.	16/22
Unsworth et al. (2019) (55)	Prospective cohort, Melbourne, Australia	To examine the predictive validity of OT-DORA Battery of the on-road test outcome for individuals with stroke.	OT-DORA Battery	On-road driving performance	3 cognitive subtests of the OT-DORA battery including MMSE, OT-DHMT and Road Law and Road Craft Test had good predictive validity of the on-road test outcome. The Road Law and Road Craft Test cut-off score is 20.5. Those with a score over 20.5 pass the on-road test, while those with less than 20.5 usually fail the on-road test.	14/22

Conditions: ABI, acquired brain injury; CVA, cerebrovascular accident; mTBI, mild traumatic brain injury; PD, parkinson’s disease; TBI, traumatic brain injury.

Tests: BD, block design; CBDI, cognitive behavioral driver’s inventory; CTT, colour trails test; DS, digit symbol; FAB, the frontal assessment battery; JLO, judgement of line orientation; MFVPT, motor free visual perception test; MMSE, mini mental state examination; MOCA, montreal cognitive assessment; MVPT, motor-free visual perception test; NDT, the neurocognitive driving test; OT- DORA Battery, occupational therapy – driver off-road assessment battery; OT–DHMT, occupational therapy-drive home maze test; P-drive, performance analysis of driving ability; PA, picture arrangement; RAVLT, rey auditory verbal learning test; RCFT, rey complex figure test; RTM, response time measures; SDMT, symbol digit modalities test; SDSA, stroke driver screening assessment; SNST, stroop neuropsychological screening test; TAP, attentional performance battery; TMT-A, trail making test part A; TMT-B, trail making test part B; UFOV, useful field of view; VFD, visual form discrimination; VRST, visual recognition slide test; VSA, visual scanning analyser; WAIS, the wechsler adult intelligence scale; WCST, wisconsin card sorting test.

### Assessment methods used to evaluate driving competence for people with ABI

The majority of the included studies investigated clinical assessment methods to evaluate driving competence following ABI (80.77%, *n* = 21) either by examining individual assessment tools or a battery of tools used in combination. Three studies used CDE (31, 32, 50) and three studies investigated use of the Performance Analysis of Driving Ability (*P*-Drive) (47–49). *P*-Drive is a measuring tool based on an analysis of the action needed to perform the activity of driving that can be used with simulated driving tests (47, 48) or on road driving test (49). Only one study reviewed the use of a simulator as a method to assess driving competence (30).

The common clinical assessment tools identified to predict on-road driving performance and inform the decision of competence to drive of individuals with ABI in this systematic review are presented in Table 3, together with variables from the International Classification of Functioning (56) measured by each tool. The clinical assessment tools in Table 3 are mostly administered using paper and pencil or *via* table top activities (such as card games), except for The Useful Field of View UFOV (57) which is a computer-based assessment. Aspects of cognitive function were assessed by all of the tools included in each study (see Table 3). Although all tools also displayed some capacity to predict on road driving performance in participants with ABI, most of the included studies highlighted that clinical assessment tools are to be used with caution, and cannot replace a test of real-life (on road) driving performance.

**Table 3:** Clinical assessment tools used in assessing competence to drive following ABI.

Tool	Functions/Abilities assessed (subtests)	Administration method and time	Studies including this tool	Conditions	ICF components covered by the tool
Useful Field of View (UFOV) (57)	Processing speed Divided attention Selective Attention	Computer-based test where the participants must detect, locate, and identify a briefly presented target Approx. 15 minutes to complete	(31, 32, 40, 43, 46, 50, 52)	Stroke TBI ABI	Impairment on the body functions level including: Mental functions Sensory functions
Wechsler Adult Intelligence Scale (WAIS) (58)	Verbal comprehension (Similarities) Perceptual reasoning (Block design) Working memory (Digit span) Processing speed (Digit symbol) General intelligence	Table top (Timing depends on the subtest administered) *Similarities* participants are presented with 2 words and required to find the conceptual link between them; *Block design* participants are required to arrange blocks in a 2 × 2 or 3 × 3 array to match a 2-dimensional picture. Based on the accuracy and time taken to completion; *Digit span* requires participants to recall a string of digits in forward or backward order; *Digit symbol* speeded test that require participants to transcode numbers into symbols	(38, 45, 51, 52)	ABI Stroke TBI MS Other	Impairment on the body functions level including: Mental functions
Trail Making Test (TMT): A and B (59)	Cognitive flexibility Motor control Perceptual complexity Visual scanning And executive function	Paper and pencil (5-10 minutes) Participants connecting letters and numbers in specific order	(35, 36, 38, 42, 45, 46, 51, 52)	Stroke TBI/ABI Dementia Parkinson’s disease Other conditions with neurocognitive impairments	Impairment on the body functions level including: Mental functions Sensory functions Neuromusculoskeletal and movement related functions
Rey Complex Figure Test (RCFT) (60)	Visuospatial recall memory Visuospatial recognition memory Response bias Processing speed Visuospatial constructional ability	Paper and pencil Participant is required to copy a complex figure and depending on the examiner preference redraw it from memory after 3 minutes for immediate recall or 30 minutes for delayed recall	(31, 32, 35, 45)	Stroke TBI Dementia Parkinson’s disease Other conditions with neurocognitive impairments	Impairment on the body functions level including: Mental functions Sensory functions
Stroke Drivers’ Screening Assessment (SDSA) (61)	Attention Executive abilities Visuo-spatial abilities Memory (Dot cancellation; Square metrics; Road sign recognition)	Table top (30 minutes) *Dot cancellation* participants are required to cross out all groups of 4 dots on a paper containing 625 groups of 3, 4, and 5 dots in a maximum time of 15 minutes.; *Square metrics* involved correctly placing 16 cards, each containing 2 vehicles traveling in different directions, on 16 squares arranged in a 4 × 4 matrix in 5 minutes; *Road sign recognition* placing the correct traffic sign from 19 available traffic signs on 1 of 12 traffic situations, depicted on cards and laid out in front of the participant, in 3 minutes.	(32–34, 38, 40, 54, 55, 62)	Stroke	Impairment on the body functions level including: Mental functions Sensory functions
Mini Mental State Exam (MMSE) (63)	General cognitive status	A questionnaire (About 10 minutes-Not timed)	(34, 38, 55)	Stroke ABI	Impairment on the body functions level including: Mental functions
Occupational Therapy Drive Home Maze test (OT-DHMT) (64)	Planning Visuo-constructional skills Executive function Decision making Attentional skills	Paper and pencil maze test subtest of OT-DORA a few seconds	(34, 55)	TBI Stroke	Impairment on the body functions level including: Mental functions Sensory functions
Road Law and Road Craft Test (65)	Assesses road law (including signs) and road craft (concerning how to drive and factors that affect this) knowledge.	Paper and pencil questioner test (Not timed) Subtest of OT-DORA	(34, 55)	TBI Stroke	Impairment on the body functions level including: Mental functions
Rey Auditory Verbal Learning Test (RAVLT) (66)	Learning Immediate memory Delayed memory	Participants listen to a list of 15 words over 5 repeats. The total number of words recalled correctly over the learning trail is recorded. Then after 30 minutes the words recalled correctly are scored for the delayed trail.	(35, 45)	Stroke TBI Dementia Parkinson’s disease Other conditions with neurocognitive impairments	Impairment on the body functions level including: Mental functions

Ortho, orthopaedical conditions; TBI, traumatic brain injuries; MS, multiple sclerosis; MCI, mild cognitive impairment; ABI, acquired brain injuries; ICF, international classification of functioning, disability and health.

Three studies reported using CDE that consists of clinical and on-road testing to inform team decision on driving competence after ABI (31, 32, 50). The clinical part of the studies (31, 32) included visual tests and neuropsychological tests such as (UFOV, Figure of Ray). Moreover, the Stroke Driver Screening Assessment SDSA components that had proven to be predictive of on road testing were also measured in (32). Similarly, the UFOV and the SDSA were used in a study (50) as part of the clinical assessment, however, this study included a simulated driving test as part of the CDE in addition to on road driving test. Findings from one study were inconclusive (31), while another (32) identified that a combination of the Figure of Ray, visual neglect and the on road testing is the best model to assess driving competence after stroke. One study (50) recommended the use of the UFOV with a driving simulator.

*P*-Drive was found to be a valid scoring method that could be used to assess driving competence for an on road test (49). While only one study undertaken in 1997, with very limited data, investigated the use of a driving simulator as an assessment method of driving competence after stroke (30).

### Outcome measures used to determine driving competency after ABI

There was no consistency in the outcome measures used to determine driving competency after ABI between the studies included in this systematic review. On-road driving performance was the predominant method to determine driving competence after ABI in 15 of the included studies. On the other hand, four studies measured driving performance on a simulator and two used a self-report method reporting driving status or accident involvement (34, 52). Two studies relied on team decision as an outcome measure (32, 50) and another study relied on re-instatement of driving licence (36). Furthermore, one study combined on-road driving performance with team decision (31) and another combined on-road testing with comprehensive hospital-based evaluation of visual, motor and perceptual functions to determine driving competency (53). The outcome variables for on-road driving performance were also reported in various ways: (i) a dichotomous scale of (pass, fail) (*n* = 9); or, (ii) a 3-point ordinal scale of either (pass, fail, requires lessons) or (pass, pass with conditions, fail) (*n* = 4). Three studies used variations of 4- or 5-point ordinal scales based on different factors. Noting the sample heterogeneity around demographic, driving history and injury related variables in addition to the outcome measures used (as outlined), a meta-analysis could not be performed.

### Driving assessment administration practices

Clinical and on-road assessments were administered by an occupational therapist, either a generalist or specialist in driving rehabilitation in 21 out of the 26 studies (80.8%) included. In 13 studies, driver instructors/evaluators were involved in the on-road assessment with the occupational therapist. Both professionals would accompany the client in a dual-brake car, where the role of the driver instructor/evaluator generally would be to ensure safety and provide the trip instructions, while the occupational therapist would evaluate the performance of driving on the road. Other medical professionals such as physicians, neurologist, neuropsychologists and psychologist administered the clinical assessments in five other studies, while trained graduate students administered the assessments in one study (33). Additionally, clients were usually referred by a physician when further assessment to determine competence to drive was needed. For example, some states and territories in Australia mandate having a medical clearance for the occupational therapist to initiate the assessment process.

## Discussion

Our systematic review of the available evidence indicates that there continues to be no consensus regarding the assessment methods for determining driving competence following ABI. A possible explanation may be the lack of high-quality research as most of the included studies were at level 4 of the Oxford Centre for Evidence-Based Medicine Levels of evidence (29). Another explanation could be linked to confounding factors that are frequently presented in the reviewed studies, such as the length of time between the injury, or the diagnosis, and patients' participation in driver assessment, or the severity of their disability which was not always reported in the studies. These confounding factors might directly or indirectly affect the driver assessment outcome. For instance, a person with a severe disability following a brain injury will likely have different outcomes when assessed for driving competence compared to someone whose abilities are less severely affected. Similarly, driver assessment outcomes could be expected to differ between earlier and later stages of recovery following brain injury. Finally, methodological limitations such as small sample size, lack of blinding, uncontrolled conditions and sample heterogeneity found in this review seem to be consistent with the findings of previous systematic reviews of driving related assessment methods focused on either TBI or stroke (2, 24, 68).

### Clinical assessments

The present systematic review identified a number of clinical assessment tools that could be used to inform decisions regarding driving competency following ABI, comparable with findings reported by Hird et al. (2014) (25). Their review emphasised that driving tests that measure multiple cognitive components of driving-related performance, including TMT-A, TMT-B, RCFT, SDSA and UFOV, have the ability to predict driving fitness after stroke (25). Furthermore, Baker et al. (2015) (34) reported TMT-A, TMT-B, MMSE, OT-DHMT, Clock Drawing Test, Road Law and Road Craft Test as the most appropriate tools to be used in an acute setting with mild TBI population.

Measuring cognitive functions appears to be a common practice when assessing fitness to drive for people with brain injuries. Processing speed, executive function, attention, visuospatial skills, memory and mental flexibility are frequently measured components of cognition that should be considered when assessing driving competency (73). Nevertheless, Novack et al. (2006) (46), argue that cognitive tests are weak predictors to the on-road assessment outcome. Therefore, clinical assessments of cognitive functions may provide assistance when considering a referral to on-road driving evaluation, however, they should not replace on-road assessment (22, 34, 73). Applying the ICF, a possible explanation is that available clinical assessments are focused on impairments in the body functions/structures, whereas driving is a complex task better assessed at activity participation level and as undertaken within relevant environmental conditions (71). The use of on-road driving performance testing is therefore supported and widely recognized as an appropriate method to determine driving competence, suggesting it merits consideration as part of the establishment of driver assessment practices in Saudi Arabia.

### On-Road assessment

An on-road driving test was the most common method used to determine driving competence in the reviewed studies and in other reviews, such as Devos et al. (2011) (24) who described the on-road test as a practical method of assessing driving performance. Most of the assessment methods investigated within the primary studies in this review were eventually compared to the outcome of an on road test to establish their predictive ability to determine driver competence. Even though the on road assessment method is recognised in many countries as a standard of relicensing after ABI, some valid methodological issues with using on-road testing were identified in this research. First, the professional assessors of the on-road test were not always blinded to the patient's data, which could result in over/under estimation of the test results. Second, although some studies used standardised test routes for all participants, it is impossible to control all test conditions including: weather, traffic density, and unexpected hazards (25, 35, 68). Third, the outcome of the on road testing remains subjective due to the lack of standardized scoring methods (31). As part of the development of on-road assessment practice within Saudi Arabia, establishing some standardized test routes that replicate the variety of road types, such as inner city heavy traffic conditions, long distance highways and dirt roads in the rural areas, will be necessary.

### Driving simulator assessment

Driving simulators may potentially provide a safe alternative option to on-road driving testing (30, 69) as they can be adjusted and manipulated to different driving scenarios that cannot easily be reproduced in a real-life on-road test. Moreover, driving simulators may provide a controlled environment for testing and/or research where participants with differing medical conditions would undergo the same testing procedure to assess their driving abilities. However, Iisufficient evidence was found to support the use of driving simulators in rehabilitation practice as a driving assessment method following ABI. Indeed, this review identified only one low quality study of the validity of simulators following stroke (30). Similar findings about the lack of research supporting the use of driving simulators following TBI were reported by Classen et al. (2009) (2). Moreover, there is wide variability in terms of hardware, software, available driving scenarios and scoring methods between simulators (18, 25), and they are also not widely available and somewhat expensive to purchase. Motion sickness is also mentioned in the literature (71), although it was not measured or raised in any of the studies eligible for this review. Use of simulators in practice therefore needs to be considered with caution.

### The performance analysis of driving ability (P-Drive)

While this review confirms previous findings regarding the lack of standardized scoring methods and consequent subjectivity of scoring used to report the outcomes of both on road testing and simulated testing (31, 71), it identified a tool developed over a series of research studies to provide a valid method for analyzing and scoring the performance of driving tasks (47–49). The P-Drive was initially developed for assessing driving performance following stroke on a high-fidelity simulator. It mainly focuses on observing the driver's actions and rating them based on an occupational perspective rather than relying on rating the underlying impairments (49). It is the only assessment tool reported in this review that is an activity and performance-based tool, not an impairment-based tool according to the ICF classifications when compared to the clinical assessment tools reported in Table 3. Using a valid and reliable scoring method, such as P-Drive would be useful to report the outcome of driving performance in simulated or an on road test.

### Comprehensive driving evaluation

It has been stated in the literature that CDE is the gold standard for assessing driving competence, although its validity is unknown (2, 51, 72). This approach combines clinical and on road assessment, then the result of all the tests are weighted to determine driving competence. In the studies included in this review, we found some similarities as they all used UFOV in the clinical section and a dual brake car for the on road test. However, many differences appeared as to what was the best model that could predict the team decision of driving competence (31, 32, 50). This is in line with previous research that found the clinical assessment, as well as on road testing, may vary between practitioners (2, 51). This method is expensive and time-consuming to apply in clinical practice and still more research is needed to validate the clinical assessment as well as on road assessment for people with ABI. However, it is a safe and fair mean to assess driving competence, since neither using clinical assessment nor on road assessment independent of each other is always sufficient (72).

### Limitations

Limitations of this review include not considering studies with languages other than English. Additionally, since the primary studies in this review were too different, this prevented the possibility of a meta-analysis (73). Because of the heterogeneity of the sample, study designs, settings, metrics, the assessment of driving competence, and the outcome measures used in each of the individual studies, these have been described in detail within the result section.

### Implications and future research in relation to Saudi Arabia

The assessment of driving competency after ABI has not been investigated in the Saudi medical literature. It is essential to understand how clinicians address their patients' need to resume driving after ABI in Saudi Arabia. Since the results of this review identified occupational therapists and physicians as those who are most frequently involved in the process of driving fitness assessment and decision making, a qualitative approach to gain the perspective of both professions about this issue would be useful. Moreover, in agreement with Almosallam et al. (2021), a collaborative approach to engage stakeholders in a national initiative to establish a practice model of driver assessment and rehabilitation based on the highest available level of evidence is recommended. As aspects of this practice model, the use of clinical assessments and on-road testing for assessing driving competence are indicated.

## Conclusion

This systematic review identified a number of assessment methods that could be used to inform the decision of fitness to drive following ABI, including clinical assessments, driving simulator assessment, and on-road test assessment. Clinical assessment of cognitive functions may be of assistance when considering the need for on-road driving performance assessment but is not a substitute for an on-road performance test when driving competence needs to be evaluated.

In Saudi rehabilitation services, clinicians should use functional measures, such as range of motion, brake reaction time, vision testing, and cognitive assessment, to identify potential areas of impairments that could affect driving competence of people with ABI. Further, the development of clinical guidelines for clinicians, especially occupational therapists, is crucial to inform recommendations regarding driving competence following ABI, so as to restore the community mobility and independence of those who are competent drivers and maximise the safety for road users in Saudi Arabia.

## Data Availability

The original contributions presented in the study are included in the article/**Supplementary Material**, further inquiries can be directed to the corresponding author/s.
