# Supplementary material for: Methods to evaluate driving competence for people with acquired brain injury (ABI): A systematic review
Source: Front Rehabil Sci. 2023 Jan 4;3:1020420. doi: 10.3389/fresc.2022.1020420 (PMC9846792; doi:10.3389/fresc.2022.1020420)
Supplement: Supplementary file 1 [file Datasheet2.docx]

Supplementary File 1:

**Search strategies:**

Database: CINHAL

| **#** | **Searches** |
| --- | --- |
| S17 | S15 AND S16 |
| S16 | (MH ”Occupational Therapy”) OR “occupational therapy” |
| S15 | S8 AND S14 |
| S14 | S9 OR S10 OR S11 OR S12 OR S13 |
| S13 | “brain injur*” |
| S12 | “Acquired brain injur*” |
| S11 | “traumatic brain injuri*” |
| S10 | (MH “Stroke”) OR *stroke* |
| S9 | (MH “Brain Injuries/RH/TH”) |
| S8 | S1 OR S2 OR S3 OR S4 OR S5 OR S6 OR S7 |
| S7 | “drive* rehab*” |
| S6 | “drive* Test*” |
| S5 | read* N2 drive* |
| S4 | fit* N2 Abilit* |
| S3 | fit* N2 Drive* |
| S2  S1 | “drive* assess*”  (MH “automobile driving”) OR “automobile driving” OR (MH ”automobile driver examination”) |

Database(s): *MEDLINE, Embase, PhsycINFO, Transport Databaes, and Emcare*

| **#** | **Searches** |
| --- | --- |
| 1 | automobile driving/ or automobile driver examination/ |
| 2 | driv* assess*.mp. [mp=title, abstract, original title, name of substance word, subject heading word, floating sub-heading word, keyword heading word, organism supplementary concept word, protocol supplementary concept word, rare disease supplementary concept word, unique identifier, synonyms] |
| 3 | (fit* adj2 Drive*).mp. [mp=title, abstract, original title, name of substance word, subject heading word, floating sub-heading word, keyword heading word, organism supplementary concept word, protocol supplementary concept word, rare disease supplementary concept word, unique identifier, synonyms] |
| 4 | (driv* adj2 Abilit*).mp. [mp=title, abstract, original title, name of substance word, subject heading word, floating sub-heading word, keyword heading word, organism supplementary concept word, protocol supplementary concept word, rare disease supplementary concept word, unique identifier, synonyms] |
| 5 | (read* adj2 Driv*).mp. [mp=title, abstract, original title, name of substance word, subject heading word, floating sub-heading word, keyword heading word, organism supplementary concept word, protocol supplementary concept word, rare disease supplementary concept word, unique identifier, synonyms] |
| 6 | driv* Test.mp. [mp=title, abstract, original title, name of substance word, subject heading word, floating sub-heading word, keyword heading word, organism supplementary concept word, protocol supplementary concept word, rare disease supplementary concept word, unique identifier, synonyms] |
| 7 | driv* rehab*.mp. [mp=title, abstract, original title, name of substance word, subject heading word, floating sub-heading word, keyword heading word, organism supplementary concept word, protocol supplementary concept word, rare disease supplementary concept word, unique identifier, synonyms] |
| 8 | 1 or 2 or 3 or 4 or 5 or 6 or 7 |
| 9 | exp Brain Injuries/rh, th [Rehabilitation, Therapy] |
| 10 | traumatic brain injur*.mp. |
| 11 | Acquired brain injur*.mp. |
| 12 | brain injur*.mp. |
| 13 | 9 or 10 or 11 or 12 |
| 14 | 8 and 13 |
| 15 | Occupational Therapy/ or Occupational Therap*.mp. |
| 16 | 14 and 1 |
